# Management of appendiceal perforation induced by a metallic pin: a rare case report and literature review

**DOI:** 10.1097/RC9.0000000000001136

**Published:** 2026-10-26

**Authors:** Yassine Kallel, Sarra Nemri, Bassem Mroua, Ghazi Laamiri, Mohamed Mongi Mighri, Hassen Touinsi

**Affiliations:** a Department of general surgery, Hospital Mohamed Taher Maamouri, Nabeul, Tunisia; b Faculty of Medicine of Tunis, University Tunis El Manar, Tunis, Tunisia

**Keywords:** appendiceal perforation, case report, diagnostic pitfall, foreign body appendicitis, metallic pin

## Abstract

**Introduction:**

Foreign body–induced appendicitis is exceptionally rare, occurring in approximately 0.0005% of cases of acute appendicitis. Diagnosis may be delayed when symptoms overlap with more common conditions, such as nephrolithiasis. We report a case of appendiceal perforation caused by an accidentally ingested metallic pin in a patient with recurrent renal colic.

**Presentation of case:**

A 30-year-old male with recurrent renal colic presented with a 4-day history of worsening right lower quadrant pain. A plain abdominal radiograph, obtained because of his urological history, unexpectedly showed a linear radiopaque foreign body in the right iliac fossa. Contrast-enhanced CT localized the metallic pin within the appendiceal lumen and demonstrated peri-appendiceal inflammatory changes with localized perforation. The patient underwent emergency open appendectomy through a McBurney incision. Intraoperatively, the pin was found penetrating the mid-portion of the appendix, with a localized serous effusion. The foreign body was safely extracted and the appendectomy was completed. Histopathology confirmed acute suppurative appendicitis with a foreign body granulomatous reaction.

**Discussion:**

This case highlights the risk of diagnostic anchoring when a patient’s history suggests an alternative diagnosis. Although the clinical presentation was already highly suggestive of acute appendicitis, CT was essential to localize the unexpected foreign body and to exclude a concurrent urological cause.

**Conclusion:**

Foreign body–induced appendiceal perforation should be considered in atypical right lower quadrant pain, particularly when imaging reveals an unexpected foreign body. The surgical strategy should be individualized according to local inflammation and foreign body characteristics to ensure patient safety.

## Introduction

Acute appendicitis remains one of the most common surgical emergencies worldwide, carrying an estimated lifetime risk of approximately 7–8%. The condition typically arises from luminal obstruction, which triggers a cascade of progressive inflammation and ischemia^[^1^]^. Perforation is a frequent and severe complication, particularly in cases of delayed presentation, and is associated with significantly increased patient morbidity^[^2^]^.

HIGHLIGHTS

- Sharp foreign bodies can cause primary appendiceal perforation via direct mechanical trauma, bypassing the classic obstructive pathway.
- Pre-existing conditions, such as nephrolithiasis, can cause diagnostic anchoring, significantly delaying the correct diagnosis of appendicitis.
- A targeted secondary anamnesis is essential when an unexpected radiopaque object is incidentally found on a plain abdominal radiograph.
- While laparoscopy is standard, an open McBurney incision may be chosen to maintain direct control of a small, sharp foreign body and reduce the risk of losing it among the bowel loops.

In contrast to this classic pathophysiological pathway, foreign body (FB)-induced appendicitis represents a rare and distinct clinical entity, accounting for approximately 0.0005% of all acute appendicitis cases^[^3^]^. While the vast majority of ingested foreign bodies transit the gastrointestinal tract uneventfully, with fewer than 1% resulting in surgical complications^[^4^]^, a small minority manages to reach the cecum and enter the appendiceal lumen. Once trapped within this blind-ended anatomical structure, the FB is unlikely to be expelled spontaneously^[^5^]^.

Numerous sporadic cases have been reported over the years, detailing appendiceal obstruction secondary to foreign bodies^[^3^]^. In most instances, the underlying mechanism closely mirrors the classic obstructive sequence. However, sharp or pointed objects pose a unique hazard: they can directly penetrate the appendiceal wall, causing a “primary perforation” that entirely bypasses the typical phases of luminal occlusion and intraluminal pressure build-up^[^6^]^.

Consequently, FB-induced appendicitis is highly challenging to diagnose, not only due to its atypical mechanism but also because it frequently mimics more common clinical presentations. In patients with confounding medical histories, such as recurrent renal colic, the initial diagnostic evaluation can easily be misdirected toward alternative etiologies. This diagnostic anchoring may delay appropriate surgical management and increase the risk of complications^[^7^]^.

This work has been reported in compliance with the SCARE 2025 criteria for surgical case reports^[^8^]^.

## Case presentation

A 30-year-old man presented to the emergency department with a 4-day history of right lower quadrant (RLQ) abdominal pain, which had intensified significantly over the preceding 24 hours. The patient’s medical history was notable for recurrent renal colic. Upon physical examination, he was febrile at 38.2°C and exhibited localized guarding and rebound tenderness in the right iliac fossa. He also reported nausea and vomiting. Laboratory investigations revealed leukocytosis (12,000/mm^3^) with neutrophilia (82%) and an elevated C-reactive protein level (60 mg/L). There was no migratory pain, and anorexia was not documented. The Alvarado score was 8/10, indicating a high probability of acute appendicitis.

Nevertheless, given the patient’s history of urolithiasis, an initial kidney-ureter-bladder radiograph was performed to rule out a recurrent kidney stone. Unexpectedly, the radiograph revealed a linear, radiopaque metallic FB lodged in the right lower quadrant (RLQ). Upon focused follow-up questioning, the patient recalled accidentally swallowing a metallic pin several days prior.

A subsequent contrast-enhanced computed tomography (CT) scan of the abdomen and pelvis confirmed the presence of the metallic FB impacted within the appendiceal lumen (Fig. 1). Associated findings included periappendiceal fat stranding and a localized fluid collection, consistent with acute appendiceal inflammation and a mid-portion perforation.

**Figure 1.**
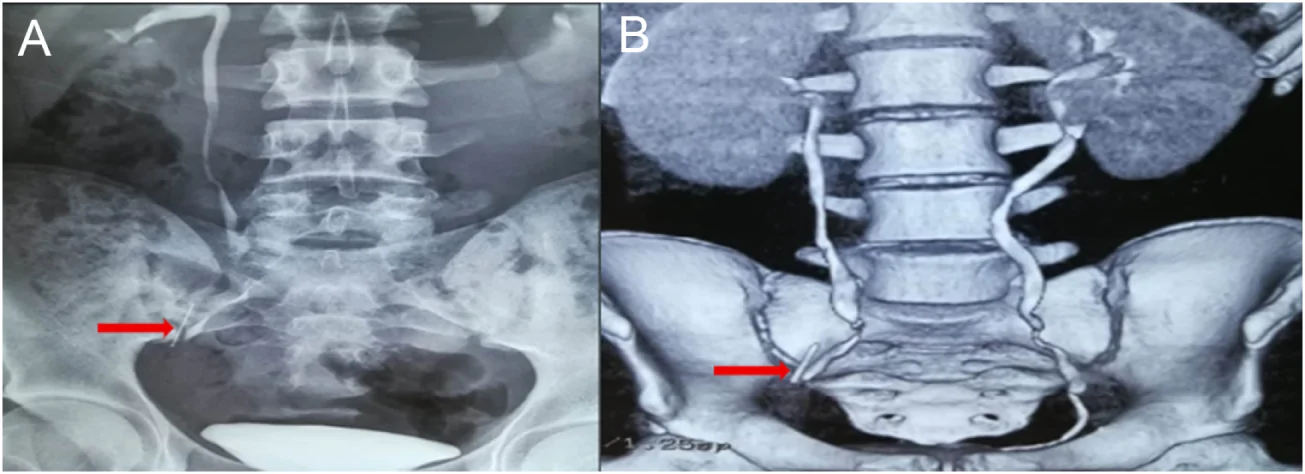
Radiological findings. (A) Post-CT abdominal radiograph demonstrates a linear radiopaque metallic foreign body (arrow) in the right iliac fossa. Residual intravenous contrast is seen in the ureter and urinary bladder after the CT examination. (B) Coronal 3D CT reconstruction reveals the extra-urinary location of the foreign body (arrow), thereby reorienting the diagnosis away from urolithiasis.

After initial resuscitation and standard preoperative preparation, the patient underwent an emergency open appendectomy through a McBurney incision, under general anesthesia with endotracheal intubation. An open approach was preferred to laparoscopy to maintain continuous control of the small, sharp pin. Displacement during insufflation was regarded as only a theoretical risk. The patient was thin, and the right iliac fossa was readily accessible through a grid-iron incision.

Intraoperative findings confirmed a perforated mid-portion of the appendix caused by the penetrating metallic pin, surrounded by a localized serous effusion (Fig. 2). Following the careful extraction of the FB, a standard appendectomy was successfully completed. No drain was placed. Perioperative management included standard intravenous antibiotic prophylaxis and postoperative analgesia, according to institutional protocol; no additional pharmacological therapy was required. The postoperative course was uneventful: oral intake was resumed on postoperative day 1, and the patient was discharged on postoperative day 2. At the 10-day follow-up, the wound had healed well and symptoms had resolved. Histopathological analysis subsequently confirmed acute suppurative appendicitis associated with a localized FB granulomatous reaction.

**Figure 2.**
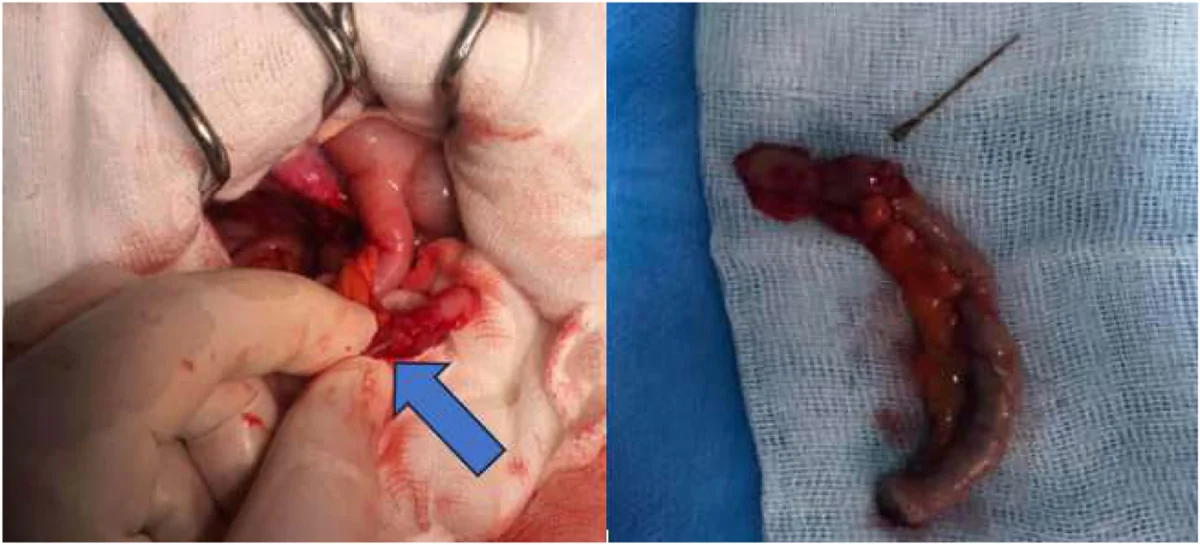
Intraoperative view: Surgical exposure via a McBurney incision demonstrating a perforated appendix with an extracted metallic pin.

## Discussion

FB-induced appendicitis is an exceptional clinical entity, accounting for approximately 0.0005% of all cases of acute appendicitis^[^3^]^. Despite its extreme rarity, it represents an important differential diagnosis of acute right lower quadrant pain. The pathogenesis generally follows the classic obstructive model: the FB obstructs the appendiceal lumen, leading to increased intraluminal pressure, ischemia, and secondary bacterial infection^[^4,5^]^. However, sharp objects such as pins or needles may cause “primary perforation” through direct mechanical trauma to the appendiceal wall, before significant intraluminal pressure develops^[^3^]^. Migration of the FB from the gastrointestinal tract into the appendix is facilitated by gravity and appendiceal stasis, particularly in the dependent pelvic position^[^9^]^.

As illustrated by this case, pre-existing medical conditions such as recurrent urolithiasis can act as significant diagnostic confounders and delay definitive care^[^10^]^. The history of renal colic directed the initial evaluation toward a simple abdominal X-ray, which unexpectedly revealed the metallic pin. While plain abdominal radiography can successfully identify certain radiopaque foreign bodies, its diagnostic utility remains limited by a low sensitivity for detecting thin or faintly opaque foreign materials, and is entirely insufficient for evaluating complex soft-tissue complications such as localized phlegmon or micro-perforations^[^11^]^.

The clinical presentation was already highly suggestive of acute appendicitis, with an Alvarado score of 8/10. The diagnosis could therefore reasonably have been established on clinical and laboratory grounds. CT was nevertheless performed for two reasons. First, the unexpected metallic FB required precise anatomical localization before surgery. Second, the history of recurrent renal colic had initially oriented the work-up toward a urological cause; cross-sectional imaging simultaneously excluded an obstructing urinary calculus and characterized the appendiceal pathology^[^11,12^]^.

Furthermore, the absence of a reported history of FB ingestion presents a well-recognized diagnostic hurdle. As observed in this case, patients rarely recall or spontaneously volunteer such information because ingestion is often completely accidental and unnoticed^[^13^]^. This diagnostic blind spot underscores the critical importance of a targeted secondary anamnesis; performing focused re-questioning immediately upon the identification of a suspicious radiopaque linear finding is an essential clinical step to prevent diagnostic anchoring and optimize surgical planning.

While the definitive treatment for FB-induced appendicitis remains appendectomy, the timing of intervention and the choice of surgical approach depend largely on the physical properties of the object and the severity of local tissue inflammation, as summarized in Table 1.

**Table 1:** Literature review of FB-induced appendicitis and surgical management.

Author (Year)	Foreign body type	Clinical presentation	Surgical approach	Key clinical findings
Klingler *et al* (1998)^[^3^]^	Various (review)	Variable	Laparotomy/laparoscopic	Landmark 100-year review, highlights high perforation risk for sharp objects.
Ozkan *et al* (2015)^[^14^]^	Shotgun pellets	Acute appendicitis	Laparotomy	Three lead pellets embedded in the lumen causing chronic intramucosal inflammation.
Mohammed *et al* (2019)^[^15^]^	Metallic nail	Acute appendicitis	Laparoscopic	Distal tip perforation with an intraluminal metallic nail.
Beh *et al* (2016)^[^13^]^	Fish bone	Acute appendicitis	Laparoscopic	Fishbone perforation mimicking acute appendicitis, handled laparoscopically.
Antonacci *et al* (2013)^[^4^]^	Marrowbone	Chronic/delayed abdominal symptoms	Laparoscopic	Extreme 15-year latency period, a large marrowbone fragment remained completely dormant inside a calcified fecaloma before triggering symptoms.
Alhasan *et al* (2026)^[^16^]^	Metallic nail	Asymptomatic	Laparoscopic	Asymptomatic metallic nail incidentally found in the appendix managed by elective laparoscopic appendectomy
Yadav *et al* (2025)^[^17^]^	Sewing machine pin	Acute appendicitis	Midline laparotomy	Situs inversus totalis in a 10-year-old boy, urgent midline laparotomy confirmed a left-sided appendix containing a 5 cm sewing machine pin
Ono *et al* (2026)^[^18^]^	Endoscopic Clip	Acute appendicitis	Midline laparotomy	Elderly woman with periappendiceal abscess caused by a migrated endoscopic clip four years after colonoscopic polypectomy, initial conservative management failed, emergency open appendectomy with partial cecectomy performed.
Present case	Metallic pin	Right lower quandrant pain mimicking renal colic	Open (McBurney)	Mid-portion perforation, localized serous effusion, diagnostic anchoring

For blunt objects, the clinical course often allows for standard urgent planning. Conversely, sharp or pointed foreign bodies with suspected or established appendiceal perforation generally warrant prompt surgical assessment and management, tailored to the clinical and anatomical circumstances in order to limit progression toward diffuse peritonitis^[^19^]^.

Laparoscopic appendectomy is widely recognized as the preferred approach for both uncomplicated and complicated acute appendicitis^[^12^]^. Several reports have also shown that ingested appendiceal foreign bodies can be managed laparoscopically^[^4,13,15^]^. However, the available evidence consists mainly of case reports and small case series, and no comparative study has established the superiority of laparoscopic or open surgery for sharp appendiceal foreign bodies. The operative strategy should therefore be individualized according to the nature and location of the FB, the local inflammatory findings, and the surgeon’s experience. In this patient, open appendectomy through a McBurney incision was an individualized decision. The principal concern was loss of the metallic pin during laparoscopic extraction. Once a small, sharp object is released from the appendix, it may disappear between small-bowel loops and become difficult to retrieve. A theoretical risk of displacement of a free-lying sharp object during pneumoperitoneum was also considered, although no published evidence specifically supports this mechanism. Finally, the patient was thin and the inflammatory process was confined to the right iliac fossa, so a McBurney incision provided adequate exposure.

This case highlights three important points. First, a patient’s past medical history can easily mislead clinicians and delay the correct diagnosis, underscoring the importance of a targeted secondary anamnesis when an unexpected finding is identified on imaging. Second, contrast-enhanced CT provides essential preoperative information in foreign-body appendicitis, even when the clinical diagnosis is already strong, by precisely localizing the object and assessing associated complications. Third, the surgical approach must remain flexible and individualized: while laparoscopy is generally preferred and has been successfully used for appendiceal foreign bodies, an open approach may be chosen for controlled extraction of a sharp object.

The primary limitation of this report is that it describes a single case, which limits the generalizability of the findings. A further limitation is that a urinalysis was not obtained, despite the history of recurrent urolithiasis.

## Conclusion

FB-induced appendiceal perforation is a rare but important diagnosis. Clinicians must maintain a high index of suspicion when classic features of acute appendicitis occur in a patient with a confounding medical history. Ultimately, the surgical strategy should be carefully individualized according to the nature of the FB, the local inflammatory findings, and the surgeon’s experience to ensure patient safety.

## Data Availability

All data generated or analyzed during this study are included in this published article.

## Patient Perspective

The patient expressed satisfaction with the surgical outcome. He acknowledged that he had not initially considered reporting the ingestion of the metallic pin, and was relieved that its identification ultimately guided appropriate and timely treatment.
